# Variants of *TSC1* are associated with developmental and epileptic encephalopathy and focal epilepsy without tuberous sclerosis: For the China Epilepsy Gene 1.0 Project

**DOI:** 10.1186/s42494-024-00189-w

**Published:** 2024-11-29

**Authors:** Nanxiang Shen, Zhihong Zhuo, Xiangyun Luo, Bingmei Li, Xuqing Lin, Sheng Luo, Zilong Ye, Pengyu Wang, Na He, Yiwu Shi, Weiping Liao

**Affiliations:** 1 Department of Neurology, Institute of Neuroscience, Key Laboratory of Neurogenetics and Channelopathies of Guangdong Province and the Ministry of Education of China, The Second Affiliated Hospital, Guangzhou Medical University, Guangzhou, 510260 China; 2 Department of Pediatrics, The First Affiliated Hospital of Zhengzhou University, Zhengzhou, 450003 China; 3 Guangzhou Medical University, Guangzhou, 511436 China

**Keywords:** *TSC1* gene, *De novo* variant, Focal epilepsy, Developmental and epileptic encephalopathy

## Abstract

**Background:**

The *TSC1* gene encodes a growth inhibitory protein hamartin, which plays a crucial role in negative regulation of the activity of mTORC1 (mechanistic target of rapamycin complex 1). *TSC1* has been associated with tuberous sclerosis complex (TSC). This study aims to investigate the association between *TSC1* variants and common epilepsy.

**Methods:**

Trio-based whole-exome sequencing was performed in epilepsy patients without acquired etiologies from the China Epilepsy Gene 1.0 Project platform. The pathogenicity of the variants was evaluated according to the American College of Medical Genetics and Genomic (ACMG) guidelines.

**Results:**

Two *TSC1 de novo* variants, including c.1498 C > T/p.Arg500* and c.2356 C > T/p.Arg786*, were identified in two patients with developmental and epileptic encephalopathy (DEE). The patients exhibited frequent seizures and neurodevelopmental delay. Additionally, we identified two heterozygous *TSC1* variants that affected four individuals with focal epilepsy from two unrelated families. The four probands did not present any typical symptom of TSC and had normal brain MRI findings. The four variants were absent in the Genome Aggregation Database (gnomAD) and were predicted to be damaging with a in silico prediction tool. Based on the ACMG guidelines, the four variants were evaluated to be “pathogenic” or “likely pathogenic”. Of the patients in the China Epilepsy Gene 1.0 Project, 22 patients carried *TSC1* variants and were diagnosed with TSC. The ratio of patients carrying *TSC1* variants with or without TSC is about 5:1.

**Conclusions:**

*TSC1* is potentially associated with common epilepsy without tuberous sclerosis.

**Supplementary Information:**

The online version contains supplementary material available at 10.1186/s42494-024-00189-w.

## Background

The *TSC1* gene (OMIM *605284) is a tumor suppressor gene encoding the growth inhibitory protein hamartin [1]. *TSC1* is ubiquitously expressed, including in the brain throughout life. Tuberous sclerosis complex 1 (TSC1) interacts with the GTPase activating protein tuberin to negatively regulate rapamycin complex 1 signaling [2]. In mice, homozygous knockout of *Tsc1* leads to embryonic growth retardation and death by embryonic day 10.5–11.5, suggesting that *TSC1* plays an important role in development [3, 4].

In humans, *TSC1* variants are associated with developmental disorders and malformations, including tuberous sclerosis complex (TSC; OMIM #191100), focal cortical dysplasia syndrome (FCD, OMIM #607341), and lymphangioleiomyomatosis (OMIM # 606690) [5–7]. Both FCD and TSC are characterized by neurodevelopmental abnormalities and are often accompanied by seizures [8, 9]. However, the association between *TSC1* variants and common epilepsy has not been determined.

In this study, we performed trio-based whole-exome sequencing in epilepsy patients without acquired etiologies. Two *TSC1 de novo* variants were identified in two patients with developmental and epileptic encephalopathy (DEE), and two co-segregating variants were identified in four patients with focal epilepsy. The four probands did not present any typical symptoms of TSC such as hypomelanotic macules, facial angiofibromas, and shagreen patches. Brain magnetic resonance imaging (MRI) was normal in all patients. The four variants were evaluated as “pathogenic” or “likely pathogenic” according to the ACMG standards and guidelines. These findings suggested that *TSC1* is potentially associated with common epilepsy without tuberous sclerosis.

## Methods

### Subjects

Epilepsy patients were enrolled through the China Epilepsy Gene 1.0 Project platform between January 2020 and December 2023. The inclusion criteria were: (1) patients without acquired causes, such as stroke, tumor, or severe perinatal injuries; (2) brain MRI showing no abnormalities of brain structure. Comprehensive clinical data of the subjects was collected, including age at recruitment, gender, seizure onset age, seizure type and frequency, response to anti-seizure medications, seizure outcome, family history, and general neurological examination results. All subjects underwent assessment of developmental and intelligence status, including motor, language, cognitive function, adaptive social skills, and performance at school or work. Twenty-four-hour video electroencephalography (EEG) monitoring data included hyperventilation, intermittent photic stimulation, open-close eyes test, and sleeping recording. The outcomes of recordings were reviewed by two certified electroencephalographers. The diagnosis of epileptic seizures and epilepsy syndromes was made in accordance with the criteria established by the Commission on Classification and Terminology of the International League Against Epilepsy (1981, 2010, 2017) [10–12]. The enrolled patients were all followed up for at least one year.

### Whole-exome sequencing and bioinformatic analyses

Peripheral blood samples were collected from the probands and their parents (trios). According to the previously established standard protocol, sequence alignment, variants calling, and variant filtering were performed [13]. An individualized protocol was used to analyze the potentially disease-causing variants. First, we prioritized the rare variants with a minor allele frequency below 0.005 in the Genome Aggregation Database (gnomAD). Then, we retained potentially pathogenic variants, including missense, initiation codon, canonical splice site, frameshift, and nonsense variants. These variants were further assessed to be damaging by in silico tools such as Mutation Taster, Combined Annotation Dependent Depletion (CADD), and fitness consequences of functional annotation (fitCons) (VarCards, http://varcards.biols.ac.cn/). Genes carrying variants with segregations, *de novo* variants, hemizygous variants, or biallelic variants were selected for further analysis. These variants represented the genetic difference between patients and normal individuals in a family and potentially explained the occurrence of disease. To validate the candidate pathogenic variants, sanger sequencing was employed. All the *TSC1* variants identified in this study were annotated based on transcript NM_000368.4.

## Results

### Identification of *TSC1* variants

**Fig. 1:**
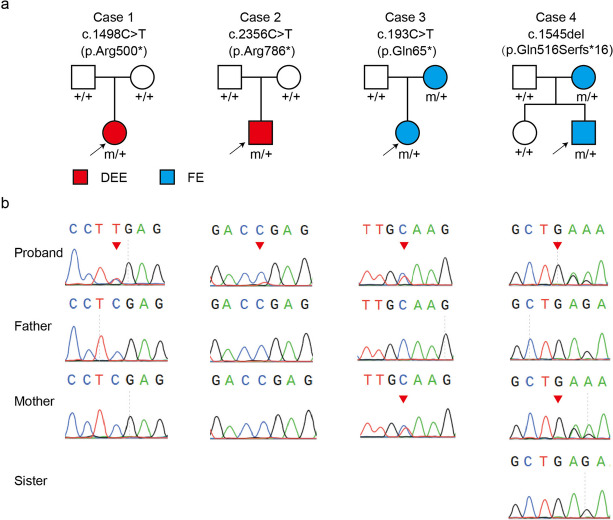
Genetic data of the four epilepsy cases with *TSC1* variants. **a** Pedigrees of the four cases carrying *TSC1* variants. Two of them suffered developmental and epileptic encephalopathy (DEE), and the other two had focal epilepsy (FE). Variants detected in each of them are shown on top. **b** DNA sequence chromatograms of the *TSC1* variants. Arrows indicate the site of mutation

Four *TSC1* variants were identified in four unrelated families with DEE or focal epilepsy (Table 1; Fig. 1a). The variants associated with DEE included two *de novo* truncation variants (c.1498 C > T/p.Arg500* and c.2356 C > T/p.Arg786*). The variants associated with focal epilepsy included one truncation variant (c.193 C > T/p.Gln65*) and one frameshift variant (c.1545del/ p.Gln516Serfs*16).

**Table 1:** Clinical features of cases with *TSC1* variants

No.	Variant (NM_000368.4)	Sex	Age (years)	Onset (years)	Seizure course	Outcome	ASMs	EEG	MRI	Development	Diagnosis
1	c.1498 C > T/ p.Arg500*	F	9 yr	4 yr	Tonic 4–6 times/day	Seizure-free for 4 years	LTG, VPA	Generalized 1.5–2.5 Hz spike-slow waves	Normal	ID	DEE
2	c.2356 C > T/ p.Arg786*	M	11 yr	5 yr	Tonic 1–3 times/day, aAb 10–15 times/day	Refractory	LEV, TPM, CNZ	Generalized and multifocal spikes. Ictal: generalized 1.5–2.5 Hz spike-slow waves	Normal	ID	DEE
3	c.193 C > T/ p.Gln65*	F	17 yr	8 yr	CPS 1–3 times/day	Seizure-free for 2 years	CBZ	Sharp-slow waves or spike-slow waves in left parietal, occipital, and temporal regions	Normal	Normal	FE
4	c.1545del/p.Gln516Serfs*16	M	26 yr	2 yr	CPS 2–3 times/month	Refractory	VPA, OXC, CNZ	Sharp waves in parietal and temporal regions	Normal	Normal	FE

*Abbreviations*: *Ab* Atypical absence, *ASMs* Anti-seizure medications, *CBZ* Carbamazepine, *CPS* Complex partial seizures, *CNZ* Clonazepam, *DEE* Developmental and epileptic encephalopathy, *EEG* Electroencephalogram, *F* Female, *FE* Focal epilepsy, *ID* Intellectual disability, *LEV* Levetiracetam, *LTG* Lamotrigine, *M* Male, *MRI* Magnetic resonance imaging, *OXC* Oxcarbazepine, *VPA* Valproate

The four variants were absent in the gnomAD database. In silico prediction tools predicted them to be damaging (Supplementary Table S1). Based on the ACMG guidelines, the four heterozygous variants were evaluated as “pathogenic” or “likely pathogenic” (Table 2).

**Table 2:** Genetic features and ACMG scorings of *TSC1* variants

Variant (NM_000368.4)	Inheritance	MAF	In silico prediction	ACMG (scoring)
c.1498 C > T/p.Arg500*	*De novo*	0	12	P (PVS1 + PS2 + PM2 + PP3)
c.2356 C > T/p.R786*	*De novo*	0	12	P (PVS1 + PS2 + PM2 + PP3)
c.193 C > T/p.Gln65*	Maternal	0	11	P (PVS1 + PM2 + PP3)
c.1545del/p.Gln516Serfs*16	Maternal	0	/	LP (PVS1 + PM3)

*Abbreviations*: *ACMG* American College of Medical Genetics and Genomics, *LP* Likely pathogenic, *P* Pathogenic, *MAF* Minor allele frequency, *PS2* *De novo* in a patient with the disease and no family history, *PM2* Absent in population databases, *PP3* Multiple lines of computational evidence support a deleterious effect on the gene/gene product, *PVS1* Null variant (nonsense, frameshift, canonical +/−1 or 2 splice sites, initiation codon, single or multi-exon deletion) in a gene where loss of function (LOF) is a known mechanism of disease

The four cases had no other pathogenic or likely pathogenic variants in other epilepsy-associated genes [14].

### Clinical features of the patients with *TSC1* variants

The summarized clinical features of the four patients with *TSC1* variants are listed in Table 1. The seizure-onset age ranged from 2 to 8 years old (median age, 4.5 years).

Case 1 and case 2 with *de novo* heterozygous variants were diagnosed with DEE. They exhibited frequent seizures with intellectual disability.

Case 1 was a girl harboring variant p.Arg500*. She experienced an atonic seizure at the age of 4 years. Later, she presented frequent tonic seizures at a frequency of 4–6 times daily. EEG of this patient showed generalized 1.5–2.5 Hz spike-slow waves. The seizures were controlled by lamotrigine and valproate.

**Fig. 2:**
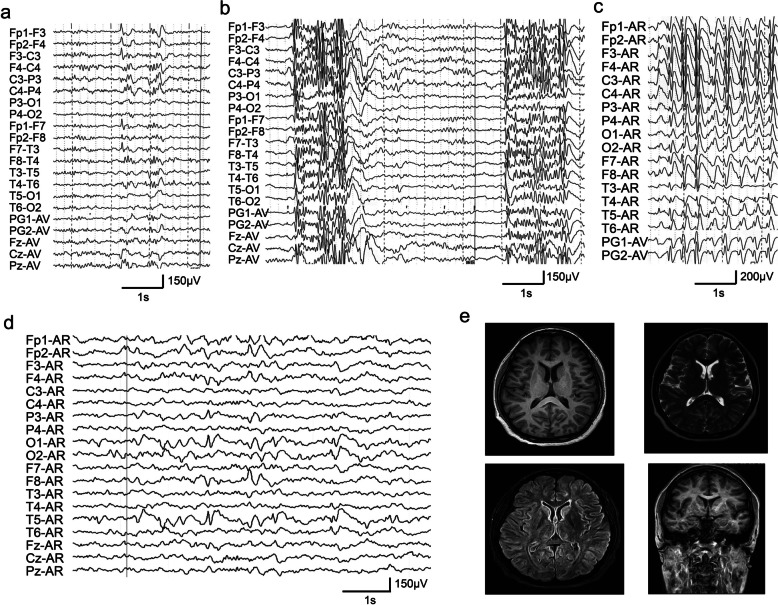
Representative EEGs and brain MRI of the cases with *TSC1* variants. **a** Interictal EEG of case 2 showing multifocal spike-waves. **b** Interictal EEG of case 2 showing generalized spike-waves. **c** Ictal EEG of case 2 showing generalized 1.5–2.5 Hz spike-slow waves. **d** Interictal EEG of case 3 showed sharp-slow waves or spike-slow waves in the left parietal, occipital, and temporal regions. **e** Brain MRI of case 3 showed normal findings

Case 2 was a boy harboring variant p.Arg786*. He initially presented tonic seizures at age of 5 years. Subsequently, he experienced multiple seizures, including tonic, atypical absence and atonic seizures at the age of 6 years. Interictal EEGs revealed generalized and multifocal discharges (Fig. 2a, b). Ictal EEG showed generalized 1.5–2.5 Hz spike-slow waves (Fig. 2c). The patient presented refractory seizures following treatment with a combination of three anti-seizure medications (ASMs).

Case 3 and case 4 inherited variants from their affected mothers. The two probands were diagnosed as focal epilepsy with focal discharges on EEG recordings. The affected mothers exhibited similar phenotypes to the probands.

The case 3 with p.Gln65* had first seizures at age of 8 years. Interictal EEGs showed sharp-slow waves or spike-slow waves in the left parietal, occipital, and temporal regions (Fig. 2d). She became seizure-free after treatment with carbamazepine. Case 4 presented refractory complex partial seizures under polytherapy.

The four patients exhibited no abnormalities in any other systems, including skin, eyes, heart, and kidneys. MRI was normal in all probands (Fig. 2e). Of the patients in the China Epilepsy Gene 1.0 project, 22 patients carried *TSC1* variants and were diagnosed with TSC. The ratio of patients carrying *TSC1* variants with TSC to those without TSC was about 5:1.

## Discussion

Variants of *TSC1* have been reported in patients with developmental disorders and malformations, such as TSC (OMIM #191100) and FCD (OMIM #607341). In this study, we identified four variants in four unrelated cases with epilepsy, including two with DEE and two with focal epilepsy. The variants associated with DEE were *de novo* variants, while those associated with focal epilepsy were inherited from the affected mothers. According to the ACMG guidelines, the four variants, which were absent from the gnomAD database and were predicted to be damaging by the majority of in silico prediction tools, were evaluated as either “pathogenic” or “likely pathogenic”. The four patients did not exhibit any of the characteristic early symptoms of TSC, and showed normal brain MRI findings. These results suggested that the *TSC1* variants are potentially associated with common epilepsy without tuberous sclerosis.

The TSC1 protein, also known as hamartin, interacts with tuberin to form the hamartin-tuberin complex, which serves as a key negative regulator of mTORC1 (mechanistic target of rapamycin complex 1) signaling. mTOR plays important roles in synaptic plasticity, brain development, and neuronal survival [15, 16]. As previously reported, TSC can be caused by various loss-of-function variants in *TSC1* [17, 18]. The diminished regulatory function of TSC1 results in the activation of the mTOR pathway, which subsequently leads to tumorigenesis and epilepsy [8, 19–21]. The severity of clinical symptoms, caused by *TSC1* variants, varies significantly among patients. The underlying mechanism of this inter-individual variability remains elusive [22–24], which may include varying severity of protein functional impairment caused by the variants, different genetic backgrounds and complex polygenic traits. In this study, we identified four *TSC1* variants in four patients with epilepsy. The four variants can result in premature termination of protein synthesis and lead to loss-of-function of TSC1 protein. None of the four patients had tuberous sclerosis, and two of them exhibited neurodevelopmental delay. This study highlighted the potential role of *TSC1* in common epilepsy.

mTOR inhibitors, such as rapamycin and everolimus, exhibit clear effectiveness in treating different tumor types in TSC. However, the significance in improving neurological symptoms is limited. Although adjunctive treatment with everolimus has demonstrated efficacy in managing focal seizures among TSC patients with epilepsy, most of the TSC patients continue to experience seizures, and many patients have shown minimal or no significant benefit from this treatment [25–27]. In this study, two patients with *TSC1* variants were seizure-free after administration with ASMs, suggesting the important role of ASMs in the treatment of patients with epilepsy caused by *TSC1* variants.

This study has several limitations. First, functional consequences of these variants were not examined and should be investigated further. Second, the number of cases with *TSC1* variants without tuberous sclerosis is limited. 

## Conclusions

*TSC1* is potentially associated with common epilepsy without tuberous sclerosis. Further studies to validate the association in large cohorts are needed in order to facilitate early genetic diagnosis and management of patients with *TSC1* variants.

## Supplementary Information

Supplementary Material 1.

## Data Availability

The data supporting the findings of this study are available from the corresponding author upon reasonable request.

## Abbreviations

ACMG: American College of Medical Genetics and Genomic
ASMs: Anti-seizure medications
DEE: Developmental and epileptic encephalopathy
EEG: Electroencephalography
FCD: Focal cortical dysplasia syndrome
MRI: Magnetic resonance imaging
TSC: Tuberous sclerosis complex
