# Supplementary material for: Variants of TSC1 are associated with developmental and epileptic encephalopathy and focal epilepsy without tuberous sclerosis: For the China Epilepsy Gene 1.0 Project
Source: Acta Epileptol. 2024 Nov 29;6:41. doi: 10.1186/s42494-024-00189-w (PMC11960315; doi:10.1186/s42494-024-00189-w)
Supplement: Supplementary file 1 — Supplementary Material 1. [file 42494_2024_189_MOESM1_ESM.docx]

Supplementary Table S1. Evaluation of the damaging effect of the *TSC1* variants identified in this study.

| Variant (NM_000368.4) | LRT | Mutation Taster | CADD | DANN | Fathmm-MKL | Eigen | GenoCanyon | fitCons | GERP++ | phyloP | phastCons | SiPhy |
| --- | --- | --- | --- | --- | --- | --- | --- | --- | --- | --- | --- | --- |
| c.193C>T/  p.Gln65* | Deleterious  (0.000) | Disease_causing  (1) | D (38) | D (0.996) | D (0.965) | D (0.861) | D (0.999) | D (0.707) | C (4.47) | C (5.521) | C (1.000) | C (14.639) |
| c.1498C>T/  p.Arg500* | Deleterious  (0.000) | Disease_causing  (1) | D (40) | D (0.998) | D (0.957) | D (0.826) | D (1.000) | D (0.732) | C (6.05) | C (4.313) | C (1.000) | C (13.060) |
| c.2356C>T/  p.Arg786* | Deleterious  (0.000) | Disease_causing  (1) | D (43) | D (0.998) | D (0.914) | D (0.748) | D (1.000) | D (0.707) | C (5.55) | C (4.286) | C (1.000) | C (18.492) |

Abbreviations: C, conserved; CADD, combined annotation dependent depletion; D, damaging; Fathmm-MKL, Functional Analysis through Hidden Markov Models–Multiple Kernels Learning; fitCons, fitness consequences of functional annotation; GERP, Genomic Evolutionary Rate Profiling; LRT, Likelihood Ratio Test; phastCons, Phylogenetic Analysis with Space/Time models conservation scoring and identification of conserved element.
